# Commencement of Lecture Term in Berkshire Medical College

**Published:** 1863-08

**Authors:** 


Commencement of Lecture Term in Berkshire Medical College. The forty-first Annual Course of Lectures in this Institution commences August 4th, and continues sixteen weeks. This is one of the oldest and most popular Medical Colleges in New England, and those who pursue their studies in this Institution will enjoy unsurpassed opportunities.

